# Endoparasite Infections in Captive Inland Bearded Dragons (*Pogona vitticeps*) in Italy

**DOI:** 10.3390/pathogens13060443

**Published:** 2024-05-23

**Authors:** Lisa Guardone, Alessandro Marigliano, Francesca Mancianti, Stefania Perrucci

**Affiliations:** Department of Veterinary Sciences, University of Pisa, Viale delle Piagge 2, 56124 Pisa, Italy; lisa.guardone@unipi.it (L.G.); ale_marigliano@libero.it (A.M.); francesca.mancianti@unipi.it (F.M.)

**Keywords:** *Pogona* spp., lizards, nematodes, protozoa, microsporidia, *Encephalitozoon pogonae*

## Abstract

The inland bearded dragon (*Pogona vitticeps*) is a lizard species commonly kept as a pet worldwide. Endoparasites are among the most important pathogens affecting bearded dragons. The aim of this study was to evaluate the endoparasites in captive *P. vitticeps* in Italy. Faecal samples from 30 *P. vitticeps* were analysed by fresh faecal smears, flotation tests, the Mini-FLOTAC technique, and a rapid immunoassay to detect *Cryptosporidium* spp. To search for microsporidia, PCR and sequencing were performed on the faecal samples. Data were statistically analysed. The overall positivity rate for endoparasites was 83.3% (25/30). The identified endoparasites were oxyurids (17/30, 56.7%), *Isosospora amphiboluri* (13/30, 43.3%), *Encephalitozoon pogonae* (4/18, 22.22%), and *Cryptosporidium* sp. (1/30, 3.33%). The positivity for protozoa was significantly higher in juveniles compared to adults. Moreover, the frequency of clinical signs was significantly higher in the positive animals. The results obtained here emphasize the importance of regular veterinary examinations of captive *P. vitticeps*, aimed at the diagnosis, treatment, and control of endoparasites. This study is one of the largest surveys on microsporidia infections in living bearded dragons, suggesting that *E. pogonae* may be widespread in this lizard.

## 1. Introduction

*Pogona vitticeps*, commonly known as the inland bearded dragon or the central bearded dragon, is an omnivorous lizard species belonging to the family Agamidae [1]. Bearded dragons (*Pogona* spp.) are widespread across Australia. Six species are currently recognized: *Pogona barbata*, *Pogona henrylawsoni*, *Pogona microlepidota*, *Pogona minor* (further subdivided into three subspecies), *Pogona nullarbor*, and *P. vitticeps* [2]. The *Pogona* spp. appearance, docility, captive hardiness, diurnal activity, and manageable size have contributed to making these animals very popular among reptile owners and breeders [3]. The central inland species (*P. vitticeps*), native to eastern and central Australia [1], has proven to be the hardiest and is consistently reproduced in captivity [2]. Indeed, this species is nowadays a popular pet worldwide, representing one of the most frequent reptiles in the pet trade and among exotic patients of veterinary clinics [1,4,5]. Accordingly, the inland bearded dragons found in Italy are also rarely imported; they are mainly bred in captivity. Captive environments should resemble natural habitats as closely as possible [6].

Parasitic infections of the digestive tract caused by nematodes and protozoa, as well as systemic infections caused by microsporidia, are included among the most important diseases affecting bearded dragons, frequently causing detrimental effects on the well-being and health of these animals [1,7,8,9]. In captive bearded dragons, intestinal nematodes and protozoa are in fact frequently recorded, with high prevalence and intensity, especially in case of poor hygiene leading to high contamination with exogenous stages of these parasites [1,7,10,11]. Moreover, additional risk factors, such as age, errors in breeding methods, concurrent diseases, and other stressing factors may predispose animals to clinical infections [1,7].

Among intestinal parasites, the coccidian species *Isosospora amphiboluri* may cause lesions to the intestinal mucosa, with consequent reduction in the absorption of nutrients and fluids, as well as diarrhoea [12,13]. This infection may be associated with disease and high mortality among juvenile bearded dragons, while in adults, *I. amphiboluri*, is generally responsible for subclinical infections [13]. Other apicomplexan protozoa are *Cryptosporidium* spp., infecting the epithelial cells of the gastrointestinal tracts of vertebrates [4]. Among bearded dragons and other reptiles, the most common species causing cryptosporidiosis are *Cryptosporidium serpentis*, *Cryptosporidium varanii* (syn. *C. saurophilum*), and *Cryptosporidium avium* [4,14,15]. Intestinal *Cryptosporidium* spp. infections in bearded dragons are often subclinical, but in massive infections and in coinfection with other parasites, cryptosporidiosis may cause enteritis associated to weight loss, anorexia, and diarrhoea, particularly in juvenile lizards [4,14,15].

Oxyurid infections are very frequently recorded among captive bearded dragons [1,16,17]. These nematodes infect the posterior gut and are generally considered of low pathogenic effects in infected reptiles [1]. However, with a high infection load, they can cause clinical diseases with diarrhoea, impaction, anorexia, severe malabsorption, and possible death [1,5,16,18].

Microsporidia are intracellular eukaryotes recently reclassified from protozoa to fungi, which can infect a wide range of vertebrate hosts, including humans, causing microsporidiosis [19]. At least three genera of microsporidia were reported in reptiles: *Encephalitozoon*, *Heterosporis,* and *Pleistophora* (syn. *Glugea*) [20]. Microsporidiosis in inland bearded dragons, reporting structures with an *Encephalitozoon*-like morphology but not identified at the species level, was first described in the USA and in Austria, as reviewed by Sokolova et al. [9]. A new species, *Encephalitozoon pogonae*, closely related to *Encephalitozoon lacertae* and *Encephalitozoon cuniculi*, was subsequently established [9]. *E. pogonae* develops primarily in macrophages within foci of granulomatous inflammation of different organs and may be responsible for fatal disseminated infection. Thus, microsporidiosis in bearded dragons is typically diagnosed post-mortem [21,22,23,24,25]. However, the presence of foci of inflammation in the gastrointestinal tract and the faecal–oral transmission allow microsporidia to be also detectable in faecal samples [22,25,26,27].

Although inland bearded dragons are common pet reptiles in Italy and in Europe, data on parasite infections in this animal are scant. Thus, the main objective of this study was the evaluation of endoparasites in *P. vitticeps* bred in captivity in Italy.

## 2. Materials and Methods

### 2.1. Faecal Samples

Faecal samples were collected from 30 individually housed pet *P. vitticeps* of both genders. The animals included 17 juvenile subjects, ≤6 months of age, and 13 adult subjects, from pet shops (18) or private owners/breeders (12). The gender was known only for adults (9 males and 4 females), but it remained undetermined for most (15/17) of the young subjects.

Freshly deposited faecal material was removed from the box of each animal and transported to the laboratory at 4 °C until the analysis, which were carried out within 24 h of collection. Clinical data compatible with endoparasite infections were also collected.

### 2.2. Parasitological Analysis

All samples were firstly qualitatively analysed by performing fresh faecal smears in saline solution to detect motile protozoa. In addition, all faecal samples (about 2 g) were examined by the flotation test and the Mini-FLOTAC technique, using saturated sodium chloride as a flotation solution (NaCl, specific gravity 1.200) for the detection and quantification of gastrointestinal nematode eggs and coccidian oocysts [28,29]. Magnifications of 100× and 400× were used to identify nematode eggs and protozoan oocysts, which were measured under a light microscope by using a micrometric eyepiece. Microscopic parasite identification was based on morphological and morphometric data [1,16,30,31,32,33,34].

Moreover, all faecal samples were examined using a commercial rapid immunoassay to search for *Cryptosporidium* spp. faecal antigens (Rida Quick^®^ *Cryptosporidium* Combi, R-Biopharm, Darmstadt, Germany).

To search for microsporidia, molecular analysis was performed on faecal samples. However, only 18/30 faecal samples were analysed, due to the scarce quantity of faecal materials available for some subjects. Total DNA was extracted from faecal samples by using a commercial kit (Kit QIAamp Fast DNA Stool Mini, QIAGEN, Hilden, Germany). Extracted DNA was subjected to PCR amplification of a 250–280 bp fragment of the 18S rRNA gene, using a primer pair (V1—CACCAGGTTGATTCTGCCTGAC; PMP2—CCTCTCCGGAACCAAACCCTG) commonly used to search for different genera of microsporidia, including *Encephalitozoon* sp. and *Enterocytozoon* sp. [35]. Amplified PCR products were sent for Sanger sequencing to an external company (Eurofins Genomics, Ebersberg, Germany). The obtained sequences were compared with those already deposited in GenBank by using the National Center for Biotechnology Information (NCBI) Basic Local Alignment Search Tool (BLAST). The obtained sequences were deposited in GenBank (accession numbers: PP824777-PP824780).

### 2.3. Statistical Analysis

The positivity rate (n. positive subjects/n. examined subjects × 100) was calculated overall and for each endoparasite. Considering the small sample size, the Fisher exact test was used to analyse differences in the positivity for endoparasites, nematodes, and protozoa between young and adult animals, and the difference in the frequency of clinical signs (lethargy, inappetence, weight and condition loss, and diarrhoea, alone or combined) between animals positive and negative for endoparasites. Values were considered significant at *p* < 0.05 and highly significant at *p* < 0.01.

## 3. Results

Among the examined animals, 12 were asymptomatic, while 18 showed diverse clinical signs, including lethargy (n = 8), anorexia (n = 8), weight and condition loss (n = 8), and diarrhoea (n = 15), alone or combined (Table 1).

The overall positivity rate for endoparasites in the examined *P. vitticeps* specimens was 83.3% (25/30). The identified endoparasites were oxyurids (17/30, 56.7%), coccidia (13/30, 43.3%), microsporidia (4/18, 22.2%), and *Cryptosporidium* sp. (1/30, 3.3%) (Table 2).

Multiple infections were found in 23.3% (7/30) of the samples. Among these animals, three were simultaneously infected by coccidia and pinworms, two were infected by coccidia, pinworms, and microsporidia, one was infected by coccidia and microsporidia, while a single bearded dragon was concurrently infected by coccidia, pinworms, and *Cryptosporidium* sp. A higher positivity rate for endoparasites was observed in young animals (94.1%, 16/17) compared to adults (61.5%, 8/13), although this difference was not statistically significant. However, while no statistical differences were observed in the positivity rate for nematodes between young and adult animals (*p* = 1.00), the positivity rate for protozoa and for coccidia considered alone was higher in juveniles compared to adults, and this difference was highly significant (*p* = 0.0008). In fact, a higher positivity rate for coccidia was observed in young animals (70.6%, 12/17) compared to adults (7.7%, 1/13), and *Cryptosporidium* was detected only in a young subject. Furthermore, a highly statistical difference (*p* = 0.0056) was found between the frequency of clinical signs in animals positive and those negative for endoparasites.

**Figure 1:**
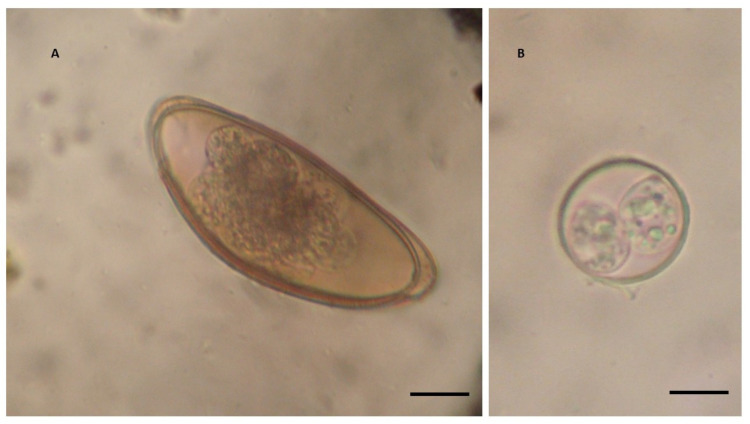
Endoparasites detected in *Pogona vitticeps* faecal samples. (**A**) Oxyurid egg. Scale bar = 22 μm. (**B**) *Isospora amphiboluri* sporulated oocyst. Scale bar = 10 μm.

Upon morphological analysis, the pinworm eggs were spindle-shaped, without polar spines, and were on average 139 μm long and 35 μm wide (range 137–141 μm × 33–38 μm) (Figure 1A). The host species, as well as the morphology and size of the eggs, are in line with those of pinworms from the family Pharyngodonidae, which are known to be capable of infecting these lizards [19,20]. The number of EPG ranged from 5 to 3910 (Table 2).

All coccidian oocysts showed a similar morphology; they were spherical or slightly subspherical, with a bilayered, smooth wall of 1.1–1.4 µm in thickness. They measured on average 24.8 μm in length and 21.8 μm in width (range 22.7–25.4 μm × 20.8–23.4 μm), with a shape index (length/width) of 1.0–1.1 (Figure 1B). Approximately 90% of the oocysts were sporulated upon the initial examination of the faecal samples. The sporulated oocysts contained two sporocysts, each containing four sporozoites, while the micropyle, oocyst residuum, and polar granules were absent. The sporocysts showed a smooth wall, a prominent Stieda body, and a sporocyst residuum and measured on average 15.8 μm in length and 11 μm in width (range 14.2–17 μm × 10.6–11.4 μm). Based on these morphological features, the oocysts were identified as *I. amphiboluri* [32,33,34] (Figure 1B).

In the positive samples, the number of coccidian oocysts ranged from 5 to 540 OPG in the quantitative analysis (Table 2).

Among microsporidia, *E. pogonae* was identified by molecular analysis. In fact, all sequences retrieved a 100% identity with other deposited sequences of this species (KR998311) by BLAST analysis.

## 4. Discussion

The results obtained in this study highlighted a high positivity rate for endoparasites in the examined captive bearded dragons. In fact, 83.3% of the animals were found positive for the presence of one or more parasitic species. These data confirm previous observations regarding the high frequency of endoparasites in bearded dragons and other pet reptiles bred in captivity. According to the literature, incorrect management practices may favour environmental contamination and, therefore, the risk of infection and of continual reinfections by many intestinal and other endoparasites with a direct life cycle [1,11,16,28,36,37]. Indeed, prevalence rates of up to about 83% for endoparasite infections have been recently reported in some parasitological studies on captive inland bearded dragons conducted in Europe [1,7,11]. However, in the study by Schmidt-Ukaj et al. [11], in which a higher prevalence was found (83%), only patients with clinical symptoms were included. The high number of juvenile animals evaluated in the present study may explain the high frequency of endoparasite infections observed. In fact, young lizards show, in general, a higher prevalence of some endoparasites, such as coccidia [1]. Moreover, although statistically not significant, data from this study showed a higher overall positivity rate for endoparasites in the examined juvenile bearded dragons (94.1%) compared to the value observed in adults (61.5%). In addition, the results obtained confirm previous data about the frequent observation of clinical signs in endoparasite-infected bearded dragons [1,7,8,11].

In this study, oxyurids, *I. amphiboluri*, *Cryptosporidium* sp., and *E. pogonae* were identified, while no motile protozoa were detected by means of fresh faecal smears, contrarily to previously reported data in bearded dragons and other lizards [1,11]. This could be due to a true negativity of the animals, limitations of this technique, mainly represented by the very little faecal material used without concentration, which can fail to detect a low number of parasites, or other factors that may not allow the microscopic recognition of protozoa moving in the field [30]. In addition, the flotation solution used in this study for coprological analysis is not suitable for the detection of some endoparasites with heavier eggs, such as trematodes [29].

Confirming previous data [1,5,7,11,34], pinworms and *I. amphiboluri* were the most frequent parasites found in this survey. A prevalence of 43.49–48.7% was previously reported for oxyurids in captive bearded dragons [1,11], in line with the data from this study (about 57%). Moreover, no statistically significant differences among juvenile and adult bearded dragons emerged from this study for oxyurid infections, and animals as young as two months old were found infected, confirming previous data in agamid lizards [1]. Agamid lizards were reported as hosts of several oxyurid species of the family Pharyngodonidae, belonging to different genera (*Parapharyngodon*, *Pharyngodon*, *Alaeuris*, and *Thelandros*) [1]. Only the genera *Pharyngodon* and *Parapharyngodon* were reported in *Pogona* spp. reptiles in previous studies [5,16,18], although it is not possible to identify the species or the genus of these parasites based only on the morphometric features of the eggs.

Oxyurid infections in lizards and other reptiles are generally considered of low pathogenic significance. However, in heavy infections, they may cause clinical disease, with anorexia, severe malabsorption, constipation, and even death [1,10,17]. Clinical infections can be observed in captive animals living in poor hygienic conditions, favouring a high environmental contamination by oxyurid eggs [1,10]. In this investigation, five bearded dragons, which were found positive for a high number of oxyurid eggs (>1200 EPG), showed clinical signs, such as lethargy, weight loss, poor body conditions, and, in some cases, also diarrhoea. Among these animals, three specimens were only infected by oxyurids, while the others were found to be coinfected with *I. amphiboluri*. On the other hand, no clinical signs were evidenced in two animals, which were found positive for a high number of oxyurid eggs.

*Isospora amphiboluri* is a coccidian species reported in both wild and captive *P. vitticeps* (inland bearded dragon), as well as in *P. barbata* (the bearded dragon) and *Ctenophorus nuchalis* (the central netted dragon) [33]. The data obtained in this study on *I. amphiboluri* infections confirmed the high frequency of this coccidian species among captive inland bearded dragons [1,11], mainly in young animals [1]. However, adult animals older than 3 years were also found infected in this study, confirming that individuals of all ages can eventually become infected by this coccidian species [1]. A high frequency of clinical symptoms, mainly diarrhoea, loss of appetite, and lethargy, was evidenced in animals found positive for *I. amphiboluri* alone, or in association with other parasites, as previously reported [12]. Indeed, of the 13 infected bearded dragons, all except one presented one or more clinical signs.

The frequency of *Cryptosporidium* infection in the examined animals (3.3%) was lower than that observed in lizards in previous studies, ranging from 10 to about 40% [1,14]. Although *Cryptosporidium* infection was reported to occur asymptomatically in inland bearded dragons and other lizards [1], the infected subjects showed intestinal symptoms characterized by diarrhoea and chronic wasting with lethargy, as previously described in *C. varanii*-infected lizards [4,38]. However, the specimen positive for *Cryptosporidium* spp. was coinfected by *I. amphiboluri* and pinworms.

The present study represents the largest survey on *E. pogonae* in the inland bearded dragon, investigating the presence of this parasite in faecal samples from living animals by molecular methods. Indeed, microsporidiosis in *P. vitticeps* was mainly reported as associated with systemic infection, and it was generally diagnosed post-mortem [21,22,23,24,25]. The first study reporting microsporidians in this lizard species was the work by Jacobson and collaborators [21], involving three animals examined post-mortem. Severe hepatic necrosis, with clusters of intracytoplasmic microorganisms in hepatocytes and in areas of necrosis, were observed in the first *P. vitticeps* analysed. Similar microorganisms were found in the renal, pulmonary, and gastric mucosal epithelial cells, enterocytes, capillary endothelial cells, and even in the ventricular ependymal cells in the brain. In the second and third necropsied lizards, analogous microorganisms were found in the macrophages present in the granulomatous inflammation of the colon, adrenal glands, and ovaries. Interestingly, no lesions were found at the gross level, but microsporidia were diagnosed only by subsequent histological examination [21]. On the contrary, necropsy examination revealed nodular granulomatous lesions in the liver and adrenal area in both cases presented by Richter et al. [22]. An intraoral mass and a mass close to the left femur were observed in the first subject described with fatal disseminated microsporidiosis in Japan, while the second subject presented nodules in the left lung [23]. Large ovarian and intracoelomic granulomas were found in captive bearded dragons in Australia [25]. Microsporidiosis was also identified by histopathology as responsible for a progressive, unilateral conjunctivitis that was not responsive to topical antibiotic or anti-inflammatory treatment in a 4-month-old female *P. vitticeps* in the USA [39].

The analysis of the literature suggests that *E. pogonae* had been previously reported as an unidentified microsporidian species or as *E. cuniculi* and may represent a common infection in bearded dragons [9]. This is supported by the results from this study. Moreover, the infection of the gastrointestinal epithelium with *Encephalitozoon* spp. [21,40,41], or the detection of unclassified microsporidian spores in faeces [26], suggests a faecal–oral route of transmission in reptiles [22]. Microsporidia spores were searched for by light microscopy, after filtration steps, in faecal samples from inland bearded dragons belonging to the colony of origin of a deceased *P. vitticeps* found to be infected by microsporidia [21]. The samples were negative, but the authors stated that if the number was low, the spores could have been missed. More recently, *E. pogonae* was detected by PCR and sequencing in cloacal swabs from a deceased bearded dragon and from other living animals from the same captive collection [25]. Thus, the use of a non-invasive diagnostic approach, such as the molecular analysis of faecal samples applied to live animals, may be useful for gaining further knowledge on the presence and diffusion of *E. pogonae* infection in this reptile species.

Of the four positive animals in this study, three were young subjects (3–4 months old), while one was a one-year-old adult. All of them were symptomatic. Specifically, a young animal showed weight loss, the other two young specimens were anorexic, and one of them was also lethargic, while the adult was anorexic. Moreover, all three young subjects showed diarrhoea. This agrees with clinical data from infected bearded dragons reported in the literature [21,22,23,25]. As regards the transmission pattern, microsporidia are common parasites of insects; a batch of crickets used to feed a *P. vitticeps* found positive for microsporidia were examined by Jacobson et al. [21], but no spores were found. Additionally, the possibility of vertical transmission among bearded dragons was also suggested [42]. Indeed, two animals with fatal microsporidiosis, in Japan, were purchased at the same facility in Tokyo and kept in different private households. The authors hypothesize the possibility that these animals had already been infected with *E. pogonae* in the facility but were asymptomatic, underlining the potential role of the reptile pet industry in the spread of *E. pogonae* [23].

## 5. Conclusions

In the captive inland bearded dragons examined in this study, a high overall frequency of endoparasite infections was observed. In addition, these infections were found to be frequently associated with clinical signs. These results emphasize the importance of including endoparasite infections in the differential diagnosis of diseases in symptomatic animals, as well as performing regular parasitological monitoring in the health screening of captive inland bearded dragons. This would allow for the implementation of appropriate control measures before parasitic infections become clinically evident in asymptomatic animals, as well as the confirmation or exclusion of parasitic infections from the possible causes of the symptoms observed. Moreover, this study is one of the largest surveys on microsporidia infections in living bearded dragons using molecular methods on faecal samples.

## Figures and Tables

**Table 1:** Origin, age, clinical signs, and positivity for endoparasites of examined 30 captive inland bearded dragons (*Pogona vitticeps*) bred in captivity in Italy.

Animal ID	Origin	Age	Clinical Signs	*Isospora* *amphiboluri*	Oxyurids	*Cryptosporidium* sp.	*Encephalitozoon pogonae*
1	P	6 months	I+D	+	+	+	−
2	P	>3 years	A	−	−	−	NE
3	P	>3 years	A	−	−	−	NE
4	S	<1 month	D	+	−	−	NE
5	S	2 months	I+L+D	+	+	−	NE
6	S	5 months	I+L+D	+	−	−	NE
7	S	2 months	I+L+D	+	−	−	−
8	S	>3 years	L+D	+	−	−	NE
9	S	>3 years	L+W+D	−	+	−	NE
10	S	>3 years	A	−	−	−	−
11	S	3 months	W+D	+	−	−	+
12	S	4 months	I+L+D	+	+	−	+
13	S	3 months	A	−	+	−	−
14	S	>3 years	W+D	−	+	−	−
15	S	3 months	I+D	+	−	−	−
16	S	3 months	I+D	+	+	−	+
17	P	6 months	W+D	+	−	−	−
18	S	5 months	A	−	+	−	−
19	P	4 months	L+W+D	+	+	−	−
20	S	>3 years	A	−	+	−	−
21	S	>3 years	W	−	+	−	−
22	S	>2 years	A	−	−	−	−
23	P	>3 years	L+W+D	−	+	−	−
24	P	1 year	I	−	−	−	+
25	P	6 months	A	−	−	−	−
26	P	5 months	A	+	+	−	NE
27	P	>3 years	W	−	+	−	NE
28	P	>3 years	A	−	+	−	NE
29	P	5 months	A	−	+	−	NE
30	S	6 months	A	−	+	−	NE

P: private owner/breeder, S: pet shop; I: inappetence; D: diarrhoea; L: lethargy; W: weight and condition loss; A: asymptomatic; +: positivity, −: negativity; NE: not examined.

**Table 2:** Positivity rate for endoparasites in faecal samples from 30 inland bearded dragons (*Pogona vitticeps*) bred in captivity in Italy.

*Pogona vitticeps*	Oxyurids	*Isospora* *amphiboluri*	*Cryptosporidium* sp.	Microsporidia
N. positive subjects/ N. examined subjects	17/30	13/30	1/30	4/18
Positivity rate	56.7%	43.3%	3.3%	22.2%
Range EPG/OPG ^1^	5–3910 EPG ^1^	5–540 OPG ^1^	n.a. ^2^	n.a. ^2^

^1^ EPG/OPG: number of eggs/oocysts per gram of faeces; ^2^ n.a.: not available.

## Data Availability

Raw data supporting the conclusions of this study are available from the authors upon request.
